# Ontarians’ Perceptions of Public Health Communications and Misinformation During the COVID-19 Pandemic: Survey Study

**DOI:** 10.2196/38323

**Published:** 2023-06-02

**Authors:** Christine Fahim, Jeanette Cooper, Suvabna Theivendrampillai, Ba' Pham, Sharon Straus

**Affiliations:** 1 Li Ka Shing Knowledge Institute St Michael's Hospital Unity Health Toronto Toronto, ON Canada

**Keywords:** misinformation, information seeking, COVID-19, trust, dissemination, health communication, risk, communication, policy maker, transmission, health emergency, age, gender, survey

## Abstract

**Background:**

Clear, accurate, and transparent risk communication is critical to providing policy makers and the public with directions to effectively implement public health strategies during a health emergency.

**Objective:**

We aimed to explore the public’s preferred sources of obtaining COVID-19 information, perceptions on the prevalence and drivers of misinformation during the pandemic, and suggestions to optimize health communications during future public health emergencies.

**Methods:**

We administered a web-based survey that included Likert scale, multiple choice and open-ended response questions to residents of Ontario, Canada. We aimed to recruit a sample that reflected population diversity with respect to age and gender. Data were collected between June 10, 2020, and December 31, 2020, and were analyzed using descriptive statistics; open-ended data were analyzed using content analysis. Subgroup analyses to explore perceptions by age and gender were conducted using ordinal regression.

**Results:**

A total of 1823 individuals participated in the survey (n=990, 54% women; n=703, 39% men; n=982, 54% aged 18-40 years; n=518, 28% aged 41-60 years; and n=215, 12% aged ≥61 years). Participants most commonly obtained COVID-19 information from local television news (n=1118, 61%) followed by social media (n=938, 51%), national or international television news (n=888, 49%), and friends and family (n=835, 46%). Approximately 55% (n=1010) of the participants believed they had encountered COVID-19–related misinformation; 70% (n=1284) of the participants reported high levels of trust in health authority websites and health care providers; 66% (n=1211) reported high levels of trust in health ministers or public health organizations. Sources perceived to be less trustworthy included friends and family, talk radio, social media, as well as blogs and opinion websites. Men were more likely to report encountering misinformation and to trust friends or family (odds ratio [OR] 1.49, 95% CI 1.24-1.79) and blogs or opinion websites (OR 1.24, 95% CI 1.03-1.50), compared to women. Compared to those aged 18-40 years, participants aged ≥41years were more likely to trust all assessed information sources, with the exception of web-based media sources, and less likely to report encountering misinformation. Of those surveyed, 58% (n=1053) had challenges identifying or appraising COVID-19 information.

**Conclusions:**

Over half of our participants perceived that they had encountered COVID-19 misinformation, and 58% had challenges identifying or appraising COVID-19 information. Gender and age differences in perceptions of misinformation and trust in information sources were observed. Future research to confirm the validity of these perceptions and to explore information-seeking patterns by population subgroups may provide useful insights on how to optimize health communication during public health emergencies.

## Introduction

Clear, accurate and transparent risk communication during health emergencies is critical to providing policy makers and the public with direction to reduce transmission through the implementation of public health strategies [1]. However, in times of uncertainty, misinformation can quickly spread, sparking public fear, distrust, and stigmatization of population subsets [2]. The World Health Organization reported that the COVID-19 pandemic was the first in history that relied heavily on social media to rapidly communicate information to the public about evolving evidence, public health precautions, and directives [3]. Yet this abundance of information resulted in an overwhelming amount of data, known as the COVID-19 “infodemic,” and led to the sharp rise of COVID-19 misinformation and disinformation [3,4]. Although misinformation and disinformation both involve the spread of false information, disinformation is false information that is purposefully disseminated to cause harm or serve a specific person, group, organization, or country agenda [3,4]. Misinformation and disinformation can be harmful and may prevent individuals from appropriately partaking in public health measures; disinformation further aims to cause distrust of groups, governments, or institutions [5]. Thus, the public’s perceptions of the quality and trustworthiness of information, regardless of its actual validity, is an important factor to explore. Susceptibility to health misinformation is complex, driven by psychological processes, levels of trust in science, and ideology [6]. Understanding public perceptions of health communication and patterns of information seeking provides important insights to developing and disseminating risk communication strategies during future health emergencies.

The purpose of this study was to explore, among those living in Ontario, Canada, preferred sources of COVID-19 health information, perceptions of encountering misinformation, levels of trust in information sources, and suggestions on optimizing public health messaging.

## Methods

### Study Design

The conduct and reporting of our survey study adhered to the Checklist for Reporting Results of Internet E-Surveys (CHERRIES) [7]. We recruited a convenience sample of residents from Ontario, Canada.

### Survey Development

Our survey was composed of 2 sections. The first section explored perceptions of stigma during the COVID-19 pandemic; these results are reported elsewhere [8]. The second section explored preferred sources of acquiring COVID-19 information during the first and second waves of the COVID-19 pandemic in Canada, perceptions of encountering misinformation during the pandemic, trust in information sources, and suggestions on how to optimize public health messaging. Questions included 5-point Likert scale, multiple choice and open-ended responses and demographic questions. Branching logic was used to further explore responses (Multimedia Appendix 1). Members of the study team’s research network piloted the survey to improve overall user experience and ensure face validity prior to dissemination. The survey was delivered in English language.

### Recruitment

We aimed to generate a sample that was evenly distributed between men and women, although we were inclusive of all gender categories. We also aimed to include a broad representation of age (≥18 years).

We conducted an open survey using several recruitment avenues and used a market organization, Canadian Viewpoint [9], to support recruitment of Ontario residents who reflected diversity in race or ethnicity. Canadian Viewpoint distributed the survey invitation to their email listservs; potential participants were those who previously agreed to receiving email communications from them. Study recruitment ads were also posted to our websites (eg, Knowledge Translation [KT] Program, Strategy for Patient-Oriented Research Evidence Alliance, KT Canada, and Unity Health Toronto) and to Twitter, LinkedIn, Kijiji, and Reddit.

### Ethics Approval

This study was approved by the Toronto Academic Health Science Network Research Ethics Board (20-092). Participants who clicked the survey were directed to a landing page, which provided an overview of the study purpose and consent information. Participation was voluntary; people who did not wish to participate were directed to close the web page or exit the browser. Responses were anonymous and survey questions were not mandatory. Participants were not compensated for participation but were provided an option to enter into a draw for the chance to win a visa gift card.

### Data Collection

Surveys were hosted through Qualtrics on our KT Program website and were also distributed via a web-based link by Canadian Viewpoint. Data were collected between June 10, 2020, and December 31, 2020.

### Data Analysis

Duplicate responses, questionnaires with less than 5% completion, or those completed by non-Ontario residents were excluded from analysis. Quantitative data were summarized using descriptive statistics; qualitative data were analyzed by using content analysis [10,11]. Responses to the questions regarding perceptions of information and trust were analyzed using ordinal regression to explore trends by gender and age. The 5-point Likert response questions regarding agreement were grouped into 3 categories (eg, “strongly disagree and disagree,” “neither agree/disagree,” and “agree and strongly agree”), and age categories were combined into 3 groups (ie, 18-40, 41-60, and ≥61 years) to facilitate analysis. Responses from gender-diverse persons, who made up less than 1% of the sample, were excluded from the regression analysis due to insufficient sample size. Responses for which the gender or age questions were not answered were excluded from the regression. Regression analyses were conducted using R statistical software (version 4.0.3; R Core Team); the R packages “foreign,” “ggplot2,” “MASS,” “Hmisc,” and “reshape2”; and RStudio software (version 2022.07.0) [12] and were overseen by a biostatistician.

## Results

### Participant Demographics

A total of 1823 individuals participated in the survey. Of them, 990 (54%) identified as women; 703 (39%) identified as men; 9 (0.49%) identified as gender fluid, transgender, gender variant, or nonbinary; and 14 (0.77%) preferred not to respond. Of 1823 participants, 982 (54%) were between 18 and 40 years of age, 518 (28%) were 41-60 years of age, and 215 (12%) were 61 years and older. Of the sample, 907 (50%) were college or university educated, 259 (14%) had some college or university education, and 336 (18%) had a postgraduate degree. Over half (n=949, 52%) of the participants worked full-time. The majority (n=1513, 83%) of the participants were Canadian citizens. Participants most commonly resided in central Ontario (n=645, 35%) and the Toronto region (n=617, 34%; Table 1).

**Table 1:** Participant demographics (N=1823).

Demographic characteristics			Values, n (%)
**Gender**			
	Woman	990 (54)	
	Man	703 (39)	
	Prefer to self-describe (gender fluid, transgender, lesbian, gender variant, and nonbinary)	9 (0.49)	
	Prefer not to say	14 (0.77)	
	No response	107 (6)	
**Age (years)**			
	18-40	982 (54)	
	41-60	518 (28)	
	≥61	215 (12)	
	No response	108 (6)	
**Race or ethnicity**			
	White	596 (33)	
	East or Southeast Asian	474 (26)	
	Black	259 (14)	
	South Asian	213 (12)	
	Others (Latinx, Middle Eastern, South American, Multiple Ethnicities, and Other)	165 (9)	
	No response	116 (6)	
**Indigenous identity**			
	Indigenous	75 (4)	
	Does not identify as Indigenous	1639 (90)	
	No response	109 (6)	
**Education level**			
	Up to 12th grade	38 (2)	
	High school or equivalent	169 (9)	
	Some college or university	259 (14)	
	College or university degree	907 (50)	
	Postgraduate degree	336 (18)	
	No response	114 (6)	
**Employment status^a^**			
	Full-time	949 (52)	
	Part-time	247 (14)	
	Caregiver	33 (2)	
	Student	144 (8)	
	Seeking work	116 (6)	
	Other	143 (8)	
	No response	116 (6)	
**Immigration status**			
	Canadian citizen (born in Canada)	921 (51)	
	Canadian citizen (born in a foreign country)	592 (32)	
	Permanent resident	142 (8)	
	Temporary resident or student visa	47 (3)	
	Other	6 (0.33)	
	No response	115 (6)	
**Place of residence**			
	Toronto region	617 (34)	
	Northern Ontario	53 (3)	
	Central Ontario	645 (35)	
	Eastern Ontario	172 (9)	
	Western Ontario	139 (8)	
	No response	197 (11)	

^a^Multiple responses per participant were possible.

### Use of Information Sources

Participants obtained COVID-19 information from local television news (n=1118, 61%); social media (n=938, 51%); national or international television news (n=888, 49%); friends and family (n=835, 46%); health authority websites (n=706, 39%); radio news (n=625, 34%); local newspapers (n=550, 30%); national or international newspapers (n=457, 25%); health care professionals (n=396, 22%); blogs or opinion websites (n=210, 12%); talk radio (n=205, 11%); and other sources (n=112, 6%), such as YouTube, scientific journals, employers and podcasts (Table 2-5).

A few differences in use of sources were noted across population subgroups. Men had greater odds of using local newspapers (odds ratio [OR] 1.44, 95% CI 1.17-1.78), international and national television news (OR 1.32, 95% CI 1.08-1.61), and talk radio (OR 1.82, 95% CI 1.35-2.48) as COVID-19 information sources and lesser odds of using health minister updates (OR 0.80, 95% CI 0.65-0.97), health authority websites (OR 0.75, 95% CI 0.60-0.91), social media (OR 0.60, 95% CI 0.49-0.74), and friends or family (OR 0.71, 95% CI 0.58-0.86) as COVID-19 information sources, compared to women (Table 6). Individuals aged 41 years and older were more likely to use radio and local television news as information sources and less likely to use social media, blogs and websites, and friends or family as sources, compared to those aged 18-40 years (Table 6).

**Table 2:** Sources of information used to obtain COVID-19 information.

Sources of information	Reported use, n (%)
Local newspapers	550 (30)
International and national newspapers	457 (25)
Local television news	1118 (61)
International and national television news	888 (49)
Radio news	625 (34)
Health minister or Public Health Ontario updates	786 (43)
Health authority websites	706 (39)
Social media	938 (51)
Friends or family	835 (46)
Health care professionals	396 (22)
Talk radio	205 (11)
Blogs and opinion websites	210 (12)
Other source (eg, YouTube and employer)	112 (6)

**Table 3:** Trust in information sources.

Sources of information	Low or very low trust, n (%)	High or very high trust, n (%)
Local newspapers	275 (15)	785 (43)
International and national newspapers	245 (13)	842 (46)
Local television news	202 (11)	1007 (55)
International and national television news	229 (13)	1021 (56)
Radio news	254 (14)	796 (44)
Health minister or Public Health Ontario updates	171 (9)	1211(66)
Health authority websites	127 (7)	1284 (70)
Social media	861 (47)	277 (15)
Friends or family	459 (25)	503 (28)
Health care professionals	111 (6)	1248 (68)
Talk radio	606 (33)	393 (22)
Blogs or opinion websites	918 (50)	242 (13)

**Table 4:** Challenges seeking COVID-19 information.

Challenges with information	Sometimes, often, or almost always, n (%)
I have had difficulty identifying the information I need	820 (45)
I have had difficulty determining the accuracy of information I found	1053 (58)
I have had difficulty sorting through conflicting information	1061 (58)
I have had difficulty making sense of information I identified (eg, unclear content or language complex)	831 (46)

**Table 5:** Perceived drivers of misinformation.

Perceived drivers of misinformation	Somewhat, a lot, or quite a lot, n (%)
Social media or community influencers	1258 (69)
International health authority	735 (40)
Federal health authority	674 (37)
Provincial health authority	649 (35)
News media	967 (53)
Academia	537 (29)
Other sources (open-ended)	328 (18)

**Table 6:** Association between reported use of information sources and demographic characteristics.

Information source	Gender, odds ratio (95% CI)^a^		Age group (years), odds ratio (95% CI)		
	Women	Men	18-40	41-60	≥61
Local newspapers	Reference	1.44 (1.17-1.78)	Reference	0.90 (0.71-1.14)	0.99 (0.71-1.36)
International and national newspapers	Reference	1.21 (0.97-1.52)	Reference	0.69 (0.53-0.88)	0.71 (0.49-1.00)
Local television news	Reference	0.86 (0.70-1.06)	Reference	1.42 (1.14-1.78)	2.09 (1.51-2.94)
International and national television news	Reference	1.32 (1.08-1.61)	Reference	1.18 (0.95-1.46)	1.75 (1.30-2.40)
Radio news	Reference	1.22 (0.99-1.50)	Reference	1.85 (1.48-2.31)	1.83 (1.34-2.48)
Health minister or Public Health Ontario updates	Reference	0.80 (0.65-0.97)	Reference	1.07 (0.86-1.32)	1.33 (0.98-1.79)
Health authority websites	Reference	0.75 (0.60-0.91)	Reference	0.89 (0.72-1.13)	0.65 (0.47-0.89)
Social media	Reference	0.60 (0.49-0.74)	Reference	0.38 (0.30-0.47)	0.20 (0.14-0.27)
Friends or family	Reference	0.71 (0.58-0.86)	Reference	0.72 (0.58-0.89)	0.50 (0.36-0.68)
Health care professionals	Reference	0.91 (0.72-1.16)	Reference	0.88 (0.67-1.15)	1.30 (0.92-1.82)
Talk radio	Reference	1.82 (1.35-2.48)	Reference	1.04 (0.74-1.46)	1.14 (0.72-1.78)
Blogs and opinion websites	Reference	1.30 (0.96-1.76)	Reference	0.47 (0.32-0.68)	0.50 (0.28-0.82)

^a^Odds ratio estimates with 95% CIs of the response of “Yes” (source used) with statements in the left column for different levels of the demographic variables.

### Trust in Information Sources

Of the participants, 70% (n=1284) and 68% (n=1248) reported high or very high levels of trust in health authority websites and health care providers, respectively. More than half of the participants reported high levels of trust in health minsters or Public Health Ontario (n=1211, 66%), international and national television news (n=1021, 56%), and local television news (n=1007, 55%). Sources seen as less trustworthy by the participants included friends and family, talk radio, social media, and blogs and opinion websites (Table 2-5).

Men were more likely to trust friends or family (OR 1.49, 95% CI 1.24-1.79) and blogs or opinion websites (OR 1.24, 95% CI 1.03-1.50) compared to women (Table 7). Participants aged 41 years and older were more likely to report increased levels of trust in almost all assessed sources of information, compared to those aged 18-40 years, with the exception of social media as well as blogs and opinion websites (Table 7).

**Table 7:** Associations between Trust in COVID-19 information sources and demographic characteristics.

How much do you trust [information source]?	Gender, odds ratio (95% CI)^a^			Age group (years), odds ratio (95% CI)		
	Women	Men	18-40		41-60	≤61
Local newspapers	Reference	0.85 (0.71-1.03)	Reference		1.42 (1.15-1.74)	1.06 (0.80-1.40)
International and national newspapers	Reference	0.91 (0.75-1.09)	Reference		1.28 (1.04-1.57)	1.16 (0.87-1.54)
Local television news	Reference	0.89 (0.73-1.07)	Reference		1.51 (1.22-1.88)	1.52 (1.12-2.06)
International and national television news	Reference	0.96 (0.79-1.16)	Reference		1.49 (1.20-1.85)	1.58 (1.17-2.15)
Radio news	Reference	1.04 (0.87-1.26)	Reference		1.63 (1.32-2.01)	1.50 (1.13-2.00)
Health minister or Public Health Ontario updates	Reference	0.82 (0.67-1.01)	Reference		1.44 (1.14-1.82)	2.14 (1.51-3.07)
Health authority websites	Reference	0.87 (0.70-1.08)	Reference		1.22 (0.97-1.56)	1.79 (1.25-2.60)
Social media	Reference	1.03 (0.86-1.24)	Reference		0.76 (0.62-0.93)	0.54 (0.40-0.72)
Friends or family	Reference	1.49 (1.24-1.79)	Reference		1.21 (0.99-1.48)	1.34 (1.01-1.77)
Health care professionals	Reference	0.97 (0.78-1.20)	Reference		1.44 (1.14-1.82)	2.95 (2.00-4.47)
Talk radio	Reference	1.05 (0.87-1.26)	Reference		1.08 (0.88-1.32)	0.98 (0.74-1.30)
Blogs and opinion websites	Reference	1.24 (1.03-1.50)	Reference		0.85 (0.69-1.05)	0.65 (0.49-0.87)

^a^Odds ratio estimates with 95% CIs of the response of “high/very high” with statements in the left column for different levels of the demographic variables.

### Challenges of Information Seeking

A total of 58% (n=1053) of the participants reported “sometimes, often, or almost always” experiencing difficulty in determining the accuracy of COVID-19 information and sorting through conflicting information; 45% (n=820) of the participants reported difficulty in identifying the COVID-19–related information they needed, and 46% (n=831) had difficulty making sense of the COVID-19 information they identified (Table 2-5). Those aged 41 years and older were less likely to report challenges with identifying or making sense of the COVID-19 information compared to those aged 18-40 years (Table 8).

**Table 8:** Association Between challenges with seeking COVID-19 information and demographic characteristics.

Challenges with information	Gender, odds ratio (95% CI)^a^			Age groups, odds ratio (95% CI)		
	Women	Men	18-40		41-60	≥61
Difficulty identifying information	Reference	1.04 (0.85-1.26)	Reference		0.65 (0.52-0.81)	0.51 (0.37-0.69)
Difficulty determining accuracy of information	Reference	0.93 (0.77-1.12)	Reference		0.71 (0.58-0.87)	0.66 (0.49-0.87)
Difficulty sorting conflicting information	Reference	0.90 (0.75-1.07)	Reference		0.69 (0.56-0.84)	0.64 (0.48-0.85)
Difficulty making sense of information	Reference	1.08 (0.89-1.30)	Reference		0.72 (0.59-0.89)	0.62 (0.46-0.83)
Other challenges	Reference	1.00 (0.79-1.28)	Reference		0.74 (0.56-0.97)	0.73 (0.49-1.06)

^a^Odds ratio estimates with 95% CIs of the response of “agree/strongly agree” with statements in the left column for different levels of the demographic variables.

### Perceptions of Misinformation

Of the study sample, 55% (n=1010) believed that they encountered misinformation “sometimes, a lot, or quite a lot.” Men were more likely to report encountering misinformation than women (OR 1.29, 95% CI 1.08-1.55). Participants aged 41 years and older were less likely to report encountering misinformation than those aged 18-40 years (aged 41-60 years: OR 0.64, 95% CI 0.52-0.78; aged ≥61 years: OR 0.36, 95% CI 0.27-0.49).

Social media and community influencers were the sources most commonly perceived by participants as the biggest drivers of misinformation, followed by news media outlets, international health authorities, federal health authorities and provincial health authorities (Table 2-5). Academia was least commonly perceived to be a source of information as compared to other sources listed in the survey (Table 2-5). Men were more likely than women to perceive all of these sources to be drivers of misinformation, with the exception of social media (Table 9). Participants 41 years and older were less likely to see all explored sources as drivers of misinformation as compared to those 18-40 years old (Table 9).

**Table 9:** Association between perceptions of misinformation drivers and demographic characteristics.

Drivers of misinformation	Gender, odds ratio (95% CI)^a^			Age groups, odds ratio (95% CI)		
	Women	Men	18-40		41-60	≥61
Social media	Reference	1.06 (0.88-1.27)	Reference		0.73 (0.60-0.89)	0.79 (0.60-1.04)
International health authority	Reference	1.45 (1.20-1.76)	Reference		0.76 (0.61-0.93)	0.48 (0.34-0.66)
Federal health authority	Reference	1.36 (1.11-1.65)	Reference		0.65 (0.52-0.80)	0.46 (0.33-0.63)
Provincial health authority	Reference	1.47 (1.20-1.79)	Reference		0.58 (0.46-0.72)	0.36 (0.26-0.52)
News media	Reference	1.46 (1.22-1.76)	Reference		0.51 (0.41-0.62)	0.42 (0.31-0.56)
Academia	Reference	1.86 (1.51-2.29)	Reference		0.64 (0.50-0.81)	0.54 (0.38-0.75)
Other source	Reference	1.68 (1.25-2.26)	Reference		1.06 (0.77-1.47)	1.21 (0.74-1.98)

^a^Odds ratio estimates with 95% CIs of the response of “agree/strongly agree” with statements in the left column for different levels of the demographic variables.

### Suggestions on Optimizing Public Health Communications

Participants believed policy makers should engage in the following actions to optimize public health communications during health emergencies: share facts and information (n=1138, 62%), educate the public to distinguish accurate from inaccurate information (n=1074, 59%), correct misperceptions (n=1075, 59%), challenge myths and stereotypes (n=878, 48%), create a list of accurate or inaccurate data sources (n=794, 44%), and use social influencers to correct misinformation (n=652, 36%).

## Discussion

### Principal Findings

The COVID-19 pandemic presented a gap between the public’s desire for immediate information and the availability of evidence-based guidance. [13]. We surveyed Ontario residents during the first and second waves of the COVID-19 pandemic in Canada to assess patterns of obtaining COVID-19 health information, trust in various information sources, and perceived exposure to misinformation.

We hypothesized that participants would turn to sources they found trustworthy to obtain COVID-19 health information; however, this was not observed. Over 50% of respondents reported encountering COVID-19–related misinformation; social media, news channels, and family or friends were perceived as significant drivers of misinformation, and participants reported low levels of trust in these sources. Despite this, 51% (n=938) reported using social media to obtain COVID-19–related information; 61% (n=1118) and 49% (n=888) used local or national and international news sources, respectively; and 46% (n=835) obtained COVID-19 information from friends and family. Conversely, sources perceived to be highly trustworthy, such as health care providers or health authorities, were less commonly sought out to obtain information, though these sources likely leveraged news and social media platforms for dissemination.

Our findings are consistent with similar studies conducted during this period of the pandemic. This includes research showing that although individuals’ level of trust typically correlates with the factual quality of that source, people consume news sources that they do not inherently trust [14].

Our disaggregated results by age and gender provide further insights on trust and use of information sources. Participants aged 18-40 years were less likely to trust assessed information sources compared to those aged ≥41 years. The 18-40 age group was also more likely to report challenges with identifying trustworthy information sources compared to participants older than 41 years. This suggests individuals older than 41 years were more likely to trust in information sources, and thereby, had an easier time obtaining and sorting through information, though we did not assess whether these perceptions correlated with data validity. Those aged 41 years or older were also less likely to report encountering misinformation, which may be attributed to their increased level of trust in information sources.

With respect to gender, men were more likely than women to report news organizations, family or friends, health authorities, and academia to be sources of misinformation; they were also more likely to turn to newspapers, television news, and talk radio for COVID-19 information compared to women. There are some data to suggest that the consumption of digital media compared to traditional media (eg, newspaper) is associated [6,15] with increased belief in misinformation. In our sample, men were more likely than women to trust digital sources of media, such as opinion websites or blogs, though we did not explore which sites or sources participants in our sample used. A limitation to our study is that we did not assess the quality of information obtained and whether the participants’ perceptions of misinformation correlated with data validity [6].

Systematic review data are inconclusive regarding the correlations between age, gender, and susceptibility to believing misinformation [6]. It is likely that susceptibility to misinformation cannot be attributed directly to a single characteristic and is rather correlated with complex processes related to analytical ability, ideology, values, and trust in information sources [6].

Still, perceptions of misinformation are important. Our study suggests that public health information in Ontario should be tailored to population subgroups, recognizing gender and age differences in perceptions of information quality and patterns of information seeking. Further, health officials and policy makers should not discount the importance of disseminating public health information via news sources perceived as less trustworthy, given that data consumption was not inherently correlated with levels of trust. Other actions suggested by participants included educating the public about misinformation, challenging myths, flagging inaccurate data sources, using social media influencers to correct misinformation, and sharing facts and information in a timely and accessible way.

### Limitations

Our survey was limited to residents in Ontario and may not be representative of experiences of individuals residing in other Canadian provinces and territories. Although we aimed to include a diverse sample of Ontario residents, our sample included more women than men, and gender-diverse persons were underrepresented in our sample and analysis. Participants were collapsed into age groups to facilitate analyses, yet disaggregation of our broad age categories (eg, 18-40 years) may provide further insights on preferred information sources and trust. Additionally, 50% of our participants had college or university degrees, and we did not disaggregate our data based on levels of education. It is possible that these demographics as well as other characteristics, such as literacy, fluency in English language, or political alignments confounded our findings as presented. Our study did not assess other important factors, including which news or television, radio, and social media channels participants were using and whether sources seen as “trusted” (eg, academics and health authorities) used these channels to disseminate health information. We also did not evaluate the quality of information that participants obtained and trusted; thus, it is possible that individuals who did not experience challenges obtaining information were in fact trusting and sharing misinformation. Finally, our survey was disseminated during the first and second COVID-19 waves in Canada; it is likely that perceptions and opinions on misinformation evolved throughout the course of the pandemic.

### Conclusions

This study describes Ontarians’ patterns of obtaining COVID-19 health information and their levels of trust in various information sources. More than half of the participants reported encountering misinformation when seeking information about COVID-19, and many reported at least one challenge with information seeking. Participants also consumed information sources that they perceived to be less trustworthy. We noted differences in trust and information seeking patterns by gender and by age. Our results suggest that health communications during public health emergencies should be tailored to account for differences in perceptions by population subgroup and should leverage a number of sources, including those perceived as more and less trustworthy.

## Appendix

Multimedia Appendix 1 Misinformation and demographic survey questions.

## Abbreviations

KT: Knowledge Translation
OR: odds ratio

## Data Availability

The data sets generated and analyzed during this study are available from the corresponding author on reasonable request.
