# Supplementary material for: Ontarians’ Perceptions of Public Health Communications and Misinformation During the COVID-19 Pandemic: Survey Study
Source: JMIR Form Res. 2023 Jun 2;7:e38323. doi: 10.2196/38323 (PMC10241226; doi:10.2196/38323)
Supplement: Multimedia Appendix 1 [file formative_v7i1e38323_app1.docx]

Appendix 1: Survey Misinformation and Demographic Questions

Disclaimer: The misinformation questions were sent simultaneously with stigma questions. For the purposes of this paper, we have only included the questions that evoked participant experiences with information, misinformation and demographic information.

**Misinformation Questions**

1. **Are you currently a resident of Ontario?**

- Yes
- No

1. **How have you been obtaining your information regarding COVID-19? Check all that apply.**

- Newspaper - Local (such as Ottawa Citizen, Toronto Star)
- Newspaper – National or international (Globe and Mail, New York Times, The Guardian)
- Local television news (e.g. Global, CTV)
- National or international television news (e.g CBC, BBC, ABC, CNN)
- Radio news
- Provincial Health Officer and Health Minister updates (e,.g. Minister Christine Elliot)
- Health authority website (such as local health authority website, Ministry of Health,
   Public Health Agency of Canada)
- Social media (e.g. Facebook, Instagram, Twitter)
- Friends or family
- Health care professional (such as family doctor or Telehealth Ontario)
- Talk radio
- Blogs/opinion websites
- Other; please specify ________________________________________________
- I don’t know

1. **What is your level of trust in these news sources?**

|  | Very Low | Low | Neutral | High | Very High |
| --- | --- | --- | --- | --- | --- |
| Newspaper - Local (such as Ottawa Citizen, Toronto Star) |  |  |  |  |  |
| Newspaper – National or international (Globe and Mail, New York Times, The Guardian) |  |  |  |  |  |
| Local television news (e.g. Global, CTV) |  |  |  |  |  |
| National or international television news (e.g. CBC, BBC, ABC, CNN) |  |  |  |  |  |
| Radio news |  |  |  |  |  |
| Provincial Health Officer and Health Minister updates (e.g. Minister Christine Elliot) |  |  |  |  |  |
| Health authority website (such as local health authority website, Ministry of Health, Public Health Agency of Canada) |  |  |  |  |  |
| Social media (e.g. Facebook, Instagram, Twitter) |  |  |  |  |  |
| Friends or family |  |  |  |  |  |
| Healthcare professional (such as family doctor or Telehealth Ontario) |  |  |  |  |  |
| Talk radio |  |  |  |  |  |
| Blogs/opinion websites |  |  |  |  |  |
| Other (please specify) |  |  |  |  |  |
| I don’t know |  |  |  |  |  |

1. **What challenges have you experienced when seeking information about COVID-19?**

|  | Almost Never | Rarely | Sometimes | Often | Almost Always |
| --- | --- | --- | --- | --- | --- |
| I have had difficulty identifying the information I need |  |  |  |  |  |
| I have had difficulty determining the accuracy of information I found |  |  |  |  |  |
| I have had difficulty sorting through conflicting information |  |  |  |  |  |
| I have had difficulty making sense of information I identified (e.g., unclear content, language complex) |  |  |  |  |  |
| I have experienced another challenge: please list |  |  |  |  |  |

1. **Have you encountered what you perceive to be COVID-19 misinformation?**

- Not at all
- A little
- Somewhat
- A lot
- Quite a lot

1. **Do you think any of the following are to blame for COVID-19 misinformation?**

|  | Not at all | A little | Somewhat | A lot | Quite a lot |
| --- | --- | --- | --- | --- | --- |
| Social media |  |  |  |  |  |
| International health authority (i.e. WHO, etc) |  |  |  |  |  |
| Federal government/federal health authority |  |  |  |  |  |
| Provincial/territorial government/provincial health authority |  |  |  |  |  |
| News media |  |  |  |  |  |
| Academia |  |  |  |  |  |
| Other, please specify |  |  |  |  |  |

1. **Why do you think social media is to blame?**

________________________________________________________________

1. **Why do you think an International Health Authority (i.e. WHO) is to blame?**

________________________________________________________________

1. **Why do you think the Federal Government or Federal Health Authority is to blame?**

________________________________________________________________

1. **Why do you think the Provincial Government or Provincial Health Authority  is to blame?**

________________________________________________________________

1. **Why do you think news media is to blame?**

________________________________________________________________

1. **Why do you think academia is to blame?**

________________________________________________________________

1. **Why do you think Other * participant entry* is to blame?**

________________________________________________________________

1. **In your opinion, who, if any, are the biggest drivers of misinformation that leads to stigma during COVID19, select all that apply.**

- Federal Health Authorities (national/international)
- Provincial/Territorial Health Authorities (national/international)
- Municipal Health Authorities (national/international)
- News media outlets
- Academia
- Social media/Community influencers
- Family/friends
- Other ________________________________________________

1. **What should policymakers do to reduce misinformation during COVID-19? Select all that apply.**

- Correct misconceptions
- Share facts and information about COVID-19
- Challenge myths and stereotypes
- Use social influencers to correct misinformation
- Create a list of inaccurate COVID-19 sources/websites
- Educate the public to distinguish accurate information from misinformation
- Other _____________________________________

1. **Do you have any other feedback, comments, or concerns regarding stigma, fear, or misinformation surrounding COVID-19 that you would like to share with us?**

________________________________________________________________

**Demographic Questions**

1. **Which best describes your current gender identity?**Note: these questions are for demographic purposes only, we will NOT identify you based on your answers; your answers will remain confidential; your data WILL NOT be shared with anyone

- Male
- Female
- Indigenous or other cultural gender identity (e.g., two-spirit)
- Other (e.g., gender fluid, non-binary) : ________________________________________________
- Prefer not to answer

1. **What is your current age?**

- 18-30
- 31-40
- 41-50
- 51-60
- 61-70
- 71-80
- 80+

1. **Do you identify as an Indigenous person?**

- Yes
- No

1. **Please select the Indigenous Identity:**

- Non-indigenous
- Metis
- First Nations
- Inuit
- Other ________________________________________________

1. **Do you identify as a ‘racialized person’? (Defined as *persons in Canada, other than indigenous peoples, who are non-Caucasian in race or non-white in colour, regardless of place of birth or citizenship*)**

- Yes
- No
- Prefer not to answer

1. **Which of the following best describes your race or ethnic group? Please check all that apply.**

- Black – African
- Black – North American
- Black – South and Central American
- Black - Afro-Carribean
- Black - Afro-European
- LatinX or Hispanic - Central American
- LatinX or Hispanic - South American
- LatinX or Hispanic - Carribean
- LatinX or Hispanic - European
- East Asian
- Southeast Asian
- South Asian
- Middle Eastern - North African
- Middle Eastern - Middle Eastern/West Asian
- White – European
- White - North American
- South American
- Other (please specify): ________________________________________________

1. **What language(s) do you speak?**

________________________________________________________________

1. **What is your current employment status? Check all that apply to your current situation.**

- Full-time
- Part-time
- Retired
- Caregiver
- Student
- Seeking work
- Other (i.e. receiving CERB, disability benefits, etc.) ________________________________________________

1. **What is the highest level of education you have completed?**

- 12th grade or less
- Graduated high school or equivalent
- Some college/university, no degree
- College/University degree
- Post-graduate degree

1. **What is your immigration status?**

- Canadian citizen (born in Canada)
- Canadian citizen (foreign born)
- Permanent resident
- Temporary resident/student visa
- Other ________________________________________________

1. **What are the first 3 digits of your postal code?**
   *Note, this is for demographic purposes only, we will NOT ask for your address and you will NOT receive any correspondence from us. Our data will not be shared with anyone.*

________________________________________________________________
